# Investigation of Serum Angiotensin-Converting Enzyme (ACE) Concentration and ACE Gene Polymorphism in Patients With SARS-CoV-2 Pneumonia Admitted to the Emergency Department

**DOI:** 10.7759/cureus.31201

**Published:** 2022-11-07

**Authors:** Tarik Goren, Atakan Yilmaz, Mehmet Uluturk, Ramazan Sabirli, Aykut Kemanci, Murat Seyit, Mert Ozen, Alten Oskay, Aylin Koseler, Ibrahim Turkcuer

**Affiliations:** 1 Department of Emergency Medicine, Pamukkale University, Denizli, TUR; 2 Emergency Department, Bakırçay University, İzmir, TUR; 3 Emergency Medicine, Tavşanlı Doc. Dr. Mustafa Kalemli State Hospital, Kütahya, TUR; 4 Department of Biophysics, Pamukkale University, Denizli, TUR

**Keywords:** sars-cov-2, pneumonia, emergency department, ace serum levels, ace polymorphism

## Abstract

Background

This study seeks to investigate the distribution of the angiotensin-converting enzyme (ACE) gene polymorphism and serum levels in patients with viral pneumonia and predict which polymorphism will lead to severe progression of the disease.

Methodology

The serum ACE levels and ACE gene polymorphisms were successfully evaluated with respect to subsequent viral pneumonia using records of 100 patients with viral pneumonia and 100 healthy controls.

Results

ACE serum concentration was statistically significantly elevated. ACE serum concentration with a cut-off value of ≥5,256.05 pg/mL had 85.3% sensitivity and 83.2% selectivity. In addition, patients with ACE genotype D/D were 0.08 times more likely to manifest severe lung involvement than those with I/I, and patients with the I/D genotype were 0.02 times more likely than their counterparts with I/I. The computed tomography findings of the patients revealed that ACE serum concentration was significantly effective in discriminating between mild and moderate-to-severe lung involvement. No significant difference was observed between the blood parameters and ACE genotype distributions.

Conclusions

I/D polymorphism likely affects the expression of the ACE gene and/or the function of the angiotensin I converting enzyme. The D/D genotype is associated with vessel wall thickness and higher blood pressure. Strong evidence was found between D/D and I/D genotypes in the patient cohort concerning genotypes and ACE serum concentration. Further analysis showed that ACE serum levels were more elevated in the D/D genotype compared to the I/D genotype in the patient cohort.

## Introduction

Viral pneumonia is a disease of great importance responsible for morbidity and mortality as well as potentially sparking worldwide pandemics. According to the World Health Organization (WHO) data, 450 million individuals are affected with pneumonia annually globally, claiming the lives of roughly 3 million people [1]. The most recent example of this situation is the severe acute respiratory syndrome coronavirus 2 (SARS-CoV-2) pandemic. Polymerase chain reaction (PCR), despite a range of limitations, proves to be a gold standard diagnostic method for confirming SARS-CoV-2 infections [2]. In addition, thoracic computed tomography (CT) stands out as the primary imaging method in diagnosing SARS-CoV-2 and assessing the potential complications with the treatment follow-up [3]. The clinical course of the disease is categorized as mild, moderate, and severe by the lung involvement in thoracic CT [4].

The SARS-CoV-2 responsible for the coronavirus pandemic is documented to use a functional receptor, called angiotensin-converting enzyme 2 (ACE-2), for entry into host cells [5]. Because ACE is expressed in the vascular endothelial bed of the lungs in healthy individuals, serum ACE concentration tends to change in diseases inducing respiratory pathology. In addition, ACE receptors, the basic components of the renin-angiotensin-aldosterone system, are highly polymorphic as genetic markers. The polymorphism of these genes generates different reflections on diseases and clinical manifestations. The ACE enzyme is encoded by the ACE gene (17q23.3 locus) comprising 26 exons and 25 introns [6]. Functional polymorphism is present in intron 16 in the form of insertion (I allele) and/or deletion (D allele) of 289 bp Alu repeat sequence (rs4646994) [7]. Alu sequences are a heterogeneous group of primate-specific interspersed repetitive DNA elements with an estimated frequency of 500,000 to 1 million copies per genome. I/D polymorphism likely affects the expression of the ACE gene and/or the function of the angiotensin I converting enzyme [8]. The D/D genotype is associated with vessel wall thickness and higher blood pressure [9]. The presence of the D allele is linked to higher ACE enzyme activity and increased expression of angiotensin II in comparison to I allele [10].

Angiotensin II level is reportedly higher in individuals with a homozygous D allele than their counterparts with a heterozygous or homozygous I allele.

This study mainly seeks to investigate the distribution of ACE gene polymorphism in patients with SARS-CoV-2 pneumonia and predict which polymorphism will lead to severe progression of the disease. In addition, we aim to clinically assess whether serum ACE concentration can act as a marker that will affect the diagnosis, mortality, and severity of the disease in these patients.

## Materials and methods

Study population

This clinical trial focusing on serum ACE concentration and ACE gene polymorphism in patients with PCR-positive SARS-CoV-2 pneumonia admitted to the emergency department was conducted in the Emergency Medicine Department of Pamukkale University over one year as of December 1, 2020. Prior to the start of the clinical procedure, the study protocol was approved by the Non-interventional Clinical Research Ethics Committee of Pamukkale University (approval number: 2020/05). A total of 200 people aged above 18, including 100 patients and 100 healthy controls, were enrolled in the study after obtaining informed consent, in accordance with the Declaration of Helsinki. This research was supported by a grant from Pamukkale University, Scientific Research Projects Fund (2020TIPF027). This study is part of a dissertation and has been published previously in the institutional thesis library [11].

Gene expression analysis

An average of 3 mL of blood was drawn into vacuum tubes through anticoagulants (K3EDTA) from all volunteers. The collected blood was stored at -20°C until the DNA isolation phase. The DNA isolation procedure was performed using the standard phenol-chloroform method. Based on Yoshida et al. (with some modifications), the PCR method with two primers was performed for the analysis of ACE polymorphism [12]. ACE gene intron 16 fragment length of 490 bp was amplified using the following primers: - forward 5′ -CTG GAG ACC ACT CCC ATC CTT TCT-3′ - reverse 5′ -GAT GTG GCC ATC ACA TTC GTC AGA T-3′. The serum samples were obtained from the blood samples collected from the patients, and serum ACE concentration was measured with the kit (human angiotensin I converting enzyme enzyme-linked immunosorbent assay kit).

Laboratory and radiological examination

The laboratory parameters, such as neutrophil-to-lymphocyte ratio (NLR), ferritin, and D-dimer values, were assessed to identify the severity of the disease. Thoracic CT severity scores were calculated by a radiologist blinded to the study, as indicated in the literature, by performing thoracic imaging on the patients [13].

Inclusion and exclusion criteria

The inclusion criteria for the patient cohort included a diagnosis of SARS-CoV-2 pneumonia and being aged over 18 years, while the only criterion for the control cohort was being healthy. The exclusion criteria that applied to both study cohorts were being under 18 years of age and refusing to participate in the study. Patients manifesting symptoms other than SARS-CoV-2 pneumonia and controls presenting signs and symptoms of any disease were also excluded from the scope of our work.

Statistical analysis

All collected data were subjected to statistical analysis using SPSS version 25 (IBM Corp., Armonk, NY, USA). The descriptive data on the sociodemographic information of the patients were presented as numbers and percentages in a tabular form. The data for continuous variables were presented as mean ± standard deviation (SD). The Kolmogorov-Smirnov value was set at p < 0.05 for normality assumptions. In addition, the Mann-Whitney U test and Kruskal-Wallis H test were performed as non-parametric tests to analyze the statistical difference, if any, between the cohorts and various biochemical parameters. The chi-square or Fisher’s exact test was performed to compare categorical variables. Spearman correlation test was used as the non-parametric test to identify the association between serum ACE concentration and various biochemical parameters. Further, receiver operating characteristic (ROC) analysis was performed on serum ACE concentration for the differential diagnosis of the patients with SARS-CoV-2 pneumonia. It was carried out to test the power of serum ACE, D-dimer, ferritin, and NLR in differentiating severe, moderate, and mild lung involvement in SARS-CoV-2 pneumonia. Finally, we performed multivariate logistic regression to see whether SARS-CoV-2 pneumonia-related factors and ACE genotypes can predict the severity (moderate-to-severe) of the disease revealed in lung CT accurately and reliably. The significance level was set at p < 0.05 for all statistical analyses.

## Results

A total of 200 eligible subjects, including 100 patients and 100 healthy controls, were recruited into the study. Our study included 102 (51%) female and 98 (49%) male subjects. The mean age of the patients was 50.17 ± 19.94 years. The ACE genotype distribution in the control and patient cohorts was statistically non-significant, while parameters such as serum ACE concentration, ferritin, NLR, and D-dimer yielded significant differences. Based on the thoracic CT findings, 68 patients had mild lung involvement, whereas 32 patients had moderate-to-severe involvement (Table 1).

**Table 1:** Sociodemographic and clinical data of the subjects. SD = standard deviation; ACE = angiotensin-converting enzyme; CT = computed tomography; NLR = neutrophil-to-lymphocyte ratio

Demographic parameters	Control (N = 100)		Patients (N = 100)		P-value
Age (years)	Median = 27	Mean ± SD = 37.07 ± 19.66	Median = 47.5	Mean ± SD = 50.17 ± 19.94	
Gender					
Female	58	58.0	44	44.0	
Male	42	42.0	56	56.0	
ACE genotype					0.712*
D/D	37	37.0	34	34.0	
I/D	45	45.0	50	50.0	
I/I	18	18.0	16	16.0	
Lung involvement on CT					
Mild	0	0	68	68.0	
Moderate-to-severe	0	0	32	32.0	
	Mean	SD	Mean	SD	
Serum ACE (pg/mL)	2,469.77	±329.06	6,064.57	±1,074.69	<0.001
Ferritin (µg/L)	70.46	±180.59	297.04	±427.35	<0.001
D-dimer (ng/mL)	46.11	±23.17	418.44	±574.49	<0.001
NLR	2.67	±3.37	5.32	±6.05	<0.001

As illustrated in Table 2, a significant difference was detected in ferritin, D-dimer, and serum ACE concentration between patients with mild and moderate-to-severe lung involvement in the thoracic CT (p = 0.001, p = 0.003, and p < 0.001, respectively). The ACE genotype distribution and NLR levels were non-significant between those with mild and moderate-to-severe involvement (p = 0.958 and p=0.255, respectively).

**Table 2:** Comparison of lung involvement with variables on CT of the patients. *: Pearson chi-square test, Mann-Whitney U test SD = standard deviation; ACE = angiotensin-converting enzyme; CT = computed tomography; NLR = neutrophil-to-lymphocyte ratio

	Lung involvement on CT		P-value
	Mild, N (%)	Moderate to severe, N (%)	
ACE genotype			
D/D	24 (70.6)	10 (29.4)	0.958*
I/D	34 (68.0)	16 (32.0)	
I/I	10 (66.7)	6 (33.3)	
	Mean ± SD	Mean ± SD	
Serum ACE (pg/mL)	6,146.14 ± 1,152.80	5,885.62 ± 869.88	<0.001
Ferritin (µg/L)	152.20 ± 141.32	607.42 ± 633.45	0.001
D-dimer (ng/mL)	315.48 ± 455.92	656.04 ± 739.88	0.003
NLR	4.17 ± 5.21	7.77 ± 7.01	0.255

Table 3 presents a significant negative correlation only between serum ACE concentration and neutrophil percentage (r = -0.206 p = 0.040) in patients with SARS-CoV-2 pneumonia.

**Table 3:** Correlation between serum ACE levels and biochemical parameters. *: The correlation is significant at the 0.05 level (Spearman correlation test); **: The correlation is significant at the 0.01 level (Spearman correlation test). ACE = angiotensin-converting enzyme; SARS-CoV-2 = severe acute respiratory syndrome coronavirus 2; WBC = white blood cell; NLR = neutrophil-to-lymphocyte ratio; CRP = C-reactive protein

		Serum ACE concentration	
		Patients with SARS-CoV-2 Pneumonia (N = 100)	Control group (N = 100)
Fever	R	-0.072	-0.210*
	P	0.481	0.036
WBC	R	-0.107	-0.039
	P	0.292	0.701
Hemoglobin	R	0.054	-0.034
	P	0.593	0.736
Neutrophil count	R	-0.139	-0.030
	P	0.169	0.763
Neutrophil percentage	R	-0.206*	0.029
	P	0.040	0.773
Lymphocyte count	R	0.073	-0.085
	P	0.472	0.400
Lymphocyte percentage	R	0.189	-0.014
	P	0.061	0.888
Platelets	R	-0.058	0.090
	P	0.571	0.373
Monocyte	R	0.074	-0.001
	P	0.469	0.993
NLR	R	-0.169	0.064
	P	0.094	0.593
CRP	R	-0.037	0.043
	P	0.721	0.673
Urea	R	0.040	0.116
	P	0.697	0.255
Creatinine	R	0.093	0.133
	P	0.362	0.187
D-dimer	R	-0.070	-0.020
	P	0.526	0.855
Ferritin	R	0.068	0.162
	P	0.593	0.120
Troponin	R	0.021	0.047
	P	0.861	0.693

As detailed in Table 4, no significant difference was observed between blood parameters and ACE genotype distributions.

**Table 4:** Comparison of ACE genotypes and biochemical parameters of patients. Kruskal-Wallis test; post-hoc = Games-Howell test. SD = standard deviation; ACE = angiotensin-converting enzyme; WBC = white blood cell; NLR = neutrophil-to-lymphocyte ratio, CRP = C-reactive protein;

	Groups	N	Mean ± SD	Kruskal-Wallis	P-value	Post-hoc
Fever (°C)	(1) D/D	34	36.88 ± 0.68	0.270	0.874	-
	(2) I/D	50	36.90 ± 0.65			
	(3) I/I	16	36.98 ± 0.74			
WBC (K/µL)	(1) D/D	34	9.49 ± 4.80	0.128	0.938	-
	(2) I/D	50	10.48 ± 6.30			
	(3) I/I	16	9.65 ± 5.19			
Hemoglobin (g/dL)	(1) D/D	34	13.59 ± 1.93	4.414	0.110	-
	(2) I/D	50	12.99 ± 2.55			
	(3) I/I	16	14.54 ± 1.52			
Neutrophil count (K/µL)	(1) D/D	34	6.69 ± 4.76	0.508	0.776	-
	(2) I/D	50	7.75 ± 6.01			
	(3) I/I	16	7.07 ± 4.67			
Neutrophil percentage (%)	(1) D/D	34	66.33 ± 15.74	0.913	0.634	-
	(2) I/D	50	68.97 ± 14.75			
	(3) I/I	16	69.83 ± 12.19			
Lymphocyte count (K/µL)	(1) D/D	34	1.98 ± 0.98	0.343	0.834	-
	(2) I/D	50	1.94 ± 1.00			
	(3) I/I	16	1.82 ± 0.76			
Lymphocyte percentage (%)	(1) D/D	34	24.78 ± 14.32	0.715	0.699	-
	(2) I/D	50	22.38 ± 12.03			
	(3) I/I	16	22.05 ± 11.91			
Platelets (K/µL)	(1) D/D	34	254.59 ± 88.7	1.434	0.488	-
	(2) I/D	50	240.70 ± 67.22			
	(3) I/I	16	229.13 ± 70.49			
Monocyte (K/µL)	(1) D/D	34	0.67 ± 0.37	1.218	0.544	-
	(2) I/D	50	0.67 ± 0.71			
	(3) I/I	16	0.64 ± 0.29			
NLR	(1) D/D	34	5.94 ± 7.51	0.727	0.695	-
	(2) I/D	50	5.32 ± 5.78			
	(3) I/I	16	4.16 ± 2.39			
CRP (mg/L)	(1) D/D	34	58.70 ± 83.86	0.109	0.947	-
	(2) I/D	50	47.14 ± 69.98			
	(3) I/I	16	59.17 ± 96.06			
Urea (mg/dL)	(1) D/D	34	35.47 ± 24.76	0.104	0.949	-
	(2) I/D	50	43.36 ± 45.31			
	(3) I/I	16	31.00 ± 14.93			
Creatinine (mg/dL)	(1) D/D	34	1.10 ± 1.56	2.507	0.286	-
	(2) I/D	50	0.91 ± 0.41			
	(3) I/I	16	0.94 ± 0.25			
D-Dimer (ng/mL)	(1) D/D	34	421.94 ± 573.05	0.466	0.792	-
	(2) I/D	50	481.97 ± 660.06			
	(3) I/I	16	250.93 ± 233.29			
Ferritin (µg/L)	(1) D/D	34	375.74 ± 493.04	1.943	0.379	-
	(2) I/D	50	240.23 ± 389.96			
	(3) I/I	16	309.72 ± 405.12			
Troponin (ng/L)	(1) D/D	34	15.36 ± 29.65	1.359	0.507	-
	(2) I/D	50	20.58 ± 29.30			
	(3) I/I	16	10.46 ± 7.98			

Strong evidence was found between the D/D and I/D genotypes in the patient cohort with respect to genotypes and serum ACE concentration (Table 5). Further analysis showed that serum ACE levels were more elevated in the D/D genotype compared to the I/D genotype in the patient cohort.

**Table 5:** Comparison of serum ACE concentration by ACE genotype groups. Mann-Whitney U test. SD = standard deviation; ACE = angiotensin-converting enzyme

	Groups	N	Mean ± SD	Z	P-value
Serum ACE concentration (pg/mL)	D/D	34	6,437.27 ± 1,307.08	2.132	0.016
	I/D	50	5,803.13 ± 871.46		
	D/D	34	6,437.27 ± 1,307.08	0.929	0.357
	I/I	16	6,091.19 ± 904.06		
	I/I	16	6,091.19 ± 904.06	-1.113	0.270
	I/D	50	5,803.13 ± 871.46		

**Figure 1:**
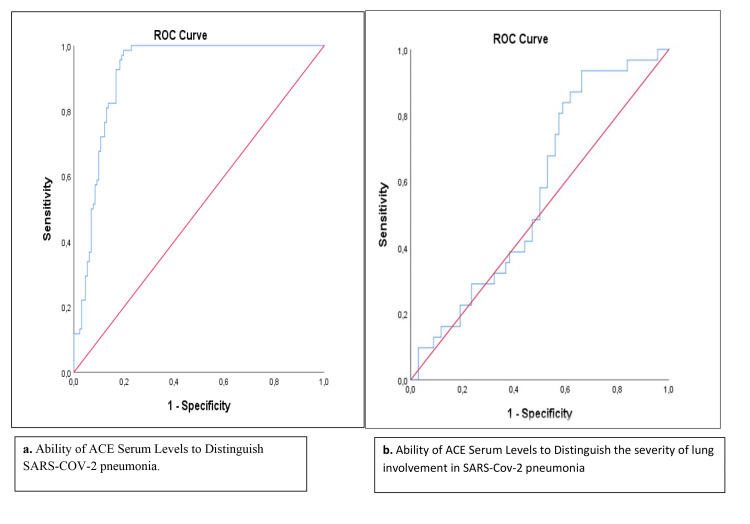
ROC curve of serum ACE concentration for SARS-CoV-2 pneumonia discrimination and computed tomography status. ROC = receiver operating characteristic, ACE = angiotensin-converting enzyme; SARS-CoV-2 = severe acute respiratory syndrome coronavirus 2

The discrimination of the presence of SARS-CoV-2 pneumonia by serum ACE concentration was statistically significant, and serum ACE concentration with a cut-off value of ≥5,256.05 pg/mL had 85.3% sensitivity and 83.2% selectivity (Table 6). ACE concentration was significant in demonstrating the presence of disease (first ROC curve) but the relationship between CT intensity and ACE concentration was not significant (second ROC curve, p = 255) (Figure 1).

**Table 6:** Comparison of AUC to assess the ability of serum ACE concentration to discriminate SARS-CoV-2 pneumonia and mild-to-severe progression on CT. AUC, 95% confidence interval. AUC = area under the curve; ACE = angiotensin-converting enzyme; SARS-CoV-2 = severe acute respiratory syndrome coronavirus 2; CT = computed tomography

	AUC	95% CI	Cut-off	Sensitivity (%)	Specificity (%)	P-value
SARS-CoV-2 pneumonia						
Serum ACE concentration (pg/mL)	0.916	0.875-0.954	≥5,256.05	85.3	83.2	<0.001
Lung involvement on CT						
Serum ACE concentration (pg/mL)	0.572	0.456-0.687	≥6,100.00	51.6	50.0	0.255

The results of the multiple logistic regression analysis in Table 7 illustrate the regression equation and significance level of the parameters. The CT imaging of the patients revealed that the likelihood of severe lung involvement increased 1.01 times with each unit increase in ferritin values and 1.00 times with each unit increase in serum ACE concentrations. In addition, patients with the ACE genotype D/D were 0.08 times more likely to manifest severe lung involvement than those with the I/I genotype, and patients with the I/D genotype were 0.02 times more likely to manifest severe lung involvement than their counterparts with the I/I genotype.

**Table 7:** Multivariate logistic regression results of SARS-CoV-2 pneumonia-related factors, ACE genotypes, and severity of lung involvement on CT. R^2^ = 0.75; -2 log likelihood = 30.360 (reference); p < 0.05 is statistically significant. ACE = angiotensin-converting enzyme; CT = computed tomography; SARS-CoV-2 = severe acute respiratory syndrome coronavirus 2

Patients with severe lung involvement on CT		
Parameters	OR (95% CI)	P-value
Ferritin	1.01 (1.00-1.02)	0.016
Serum ACE	1.00 (0.99-1.01)	0.014
ACE genotype (I/I)		0.090
D/D	0.08 (0.00-0.78)	0.039
I/D	0.02 (0.01-0.74)	0.034

## Discussion

This clinical trial was conducted among viral pneumonia patients diagnosed with SARS-CoV-2 during the pandemic. Our findings mainly indicate that the ACE genotype distribution of patients with SARS-CoV-2 pneumonia did not differ significantly from healthy controls. However, ferritin, NLR, D-dimer, and serum ACE concentration were markedly elevated in the patient cohort. Regarding the clinical outcomes, the disease progressed more severely in those with the D/D genotype in the patient population, and lung involvement became more severe as serum ACE concentration increased.

In general, the distribution of ACE genotypes in healthy populations is characterized by 25% insertion/insertion (I/I), 50% insertion/deletion (I/D), and 25% deletion/deletion (D/D) [14]. In our study, ACE genotypes were distributed as 17% I/I, 47.5% I/D, and 35.5% D/D, which is consistent with data on the distribution in the Caucasian race. In other words, no significant variation was evident between the observed and expected genotype distribution. Furthermore, we revealed that ACE gene polymorphism exerted no pronounced effect between controls and patients in relation to developing SARS-CoV-2 pneumonia and lung involvement (mild or moderate to severe) induced by the disease. Among patients with severe lung involvement, those with the ACE genotype D/D suffered 0.08 times more severe lung involvement than those with the I/I genotype, while patients with the I/D genotype had 0.02 times more severe lung involvement than their counterparts with the I/I genotype. In parallel with our results, earlier reports have demonstrated that the D/D genotype is indicative of poor prognosis for different disease groups [15,16].

Serum ACE concentration reportedly increases in SARS-CoV-2 pneumonia. Indeed, ACE-2 level is claimed to function as a new underlying biomarker for SARS-CoV-2 [17]. In the D/D genotype, endothelial dysfunction occurs due to increased serum ACE concentration, which may catalyze various ischemic events [18]. Regarding our findings, serum ACE concentrations were elevated in patients with SARS-CoV-2 pneumonia than in controls, and the greatest elevation was detected in the D/D genotype. In the differential diagnosis of SARS-CoV-2 pneumonia, we found that serum ACE concentration with a cut-off value of ≥5,256.05 pg/mL yielded significant results in differentiating the disease. Moreover, the elevation in serum ACE concentration was parallel to the lung involvement in patients manifesting severe involvement on CT. Likewise, serum ACE concentration in vascular and coronary artery diseases has been documented to be more elevated in the D/D genotype than in its I/I counterpart, and this concentration heightens the risk of mortality in coronary artery disease [19]. Our findings concerning the association between serum ACE concentration and D/D genotype in the context of SARS-CoV-2 disease broadly support the findings of other studies in this area which show that SARS-CoV-2 patients with the D/D genotype had the highest serum ACE concentration [20]. In addition, pulmonary embolism is established to be at an increased rate in those with D/D polymorphism during SARS-CoV-2 infection [21]. A clear association exists between the frequency of ACE D/D gene polymorphism and both the prevalence and mortality rates of SARS-CoV-2 [22]. Low ACE D/D genotype frequency and high ACE I/I genotype frequency observed in Asian populations are linked with low SARS-CoV-2 mortality rates in these countries [23]. Some evidence implicates the association between ACE D/D genotype and increased mortality risk for SARS-CoV-2 [24]. Previous literature reports suggest that populations with high D allele frequency in ACE genotyping have a higher mortality rate in SARS-CoV-2 and experience more severe progression, as shown in our study.

Blood lymphocyte count <800 K/µl, CRP >40 mg/L, ferritin >500 µg/L, D-dimer >1,000 ng/mL, high NLR, and high troponin are poor prognostic factors [25]. In our study, D-dimer, one of the markers of thrombosis, was approximately 10 times higher in patients with SARS-CoV-2 pneumonia than in controls. Besides, D-dimer levels were roughly two times higher in patients with severe lung involvement than those with mild involvement. In particular, SARS-CoV-2 pneumonia with severe lung involvement pose an increased risk of thrombosis. In a seminal study, 26 consecutive SARS-CoV-2 patients were screened for venous thromboembolism, and the D-dimer level was established as >1,000 ng/mL in all cases [26]. In another study, the relationship between D-dimer level and CT severity score was investigated in 86 patients with SARS-CoV-2 pneumonia. Patients with D-dimer levels >700 ng/mL had a higher rate of mechanical ventilation and four patients had pulmonary thromboembolism in the study whose D-dimer levels were all above 700 ng/mL [27].

Our findings indicate that lung involvement ran parallel to the elevation in ferritin level in patients with severe lung involvement. A clinical investigation conducted among SARS-CoV-2 patients in Israel suggested that patients with moderate-to-severe clinical status had significantly higher ferritin levels than those with mild status, and those with severe progression had markedly higher ferritin values than their counterparts with moderate progression [28]. There was also a remarkable association between elevated levels of serum ferritin and the development of acute respiratory distress syndrome in SARS-CoV-2 disease [29].

Another aspect deserving attention in our study is that NLR values were two times higher in patients with SARS-CoV-2 pneumonia than in healthy controls. Moreover, NLR level was approximately two times higher in patients with moderate-to-severe lung involvement compared to those with mild involvement. There is also mounting clinical evidence suggesting that NLR values were increased in patients diagnosed with SARS-CoV-2, as supported by our findings [30].

The major limitation of our study is that the effect of gene polymorphism on geographical or racial changes might be investigated through multicenter studies and a larger patient population. Mutation of the SARS-CoV-2 virus over the course of the pandemic may also have influenced our findings.

## Conclusions

Serum ACE concentration cut-off values of ≥5,256.05 pg/mL can make the differential diagnosis of SARS-CoV-2 pneumonia possible. It is significantly more elevated in SARS-CoV-2 patients confirmed by RT-PCR and can be utilized as a biomarker in SARS-CoV-2 pneumonia. The substantial elevation in serum ACE concentration exacerbates the prognosis of the disease in patients with severe lung involvement. The difference in ACE genotype exerts no effect on the transmission of the disease, though it progresses more severely in individuals with the D/D genotype. Serum ACE concentration was higher in patients with D/D genotypes than with other genotypes during the disease process. The degree of lung involvement is observed to aggravate with the elevation in serum ACE concentration in individuals with the D/D genotype. D-dimer, NLR, and ferritin levels, which are the underlying indicators of poor prognosis, might be suggestive of the severity of lung involvement.
